# Dual Sensory Impairment in Older Adults Increases the Risk of Mortality: A Population-Based Study

**DOI:** 10.1371/journal.pone.0055054

**Published:** 2013-03-04

**Authors:** Bamini Gopinath, Julie Schneider, Catherine M. McMahon, George Burlutsky, Stephen R. Leeder, Paul Mitchell

**Affiliations:** 1 Centre for Vision Research, Department of Ophthalmology and Westmead Millennium Institute, University of Sydney, New South Wales, Australia; 2 Menzies Centre for Health Policy, University of Sydney, Sydney, New South Wales, Australia; 3 HEARing Co-operative Research Centre, University of Melbourne, Melbourne, Victoria, Australia; 4 Centre for Language Sciences, Linguistics Department, Macquarie University, Sydney, New South Wales, Australia; University of California, San Francisco, United States of America

## Abstract

Although concurrent vision and hearing loss are common in older adults, population-based data on their relationship with mortality is limited. This cohort study investigated the association between objectively measured dual sensory impairment (DSI) with mortality risk over 10 years. 2812 Blue Mountains Eye Study participants aged 55 years and older at baseline were included for analyses. Visual impairment was defined as visual acuity less than 20/40 (better eye), and hearing impairment as average pure-tone air conduction threshold greater than 25 dB HL (500–4000 Hz, better ear). Ten-year all-cause mortality was confirmed using the Australian National Death Index. After ten years, 64% and 11% of participants with DSI and no sensory loss, respectively, had died. After multivariable adjustment, participants with DSI (presenting visual impairment and hearing impairment) compared to those with no sensory impairment at baseline, had 62% increased risk of all-cause mortality, hazard ratio, HR, 1.62 (95% confidence intervals, CI, 1.16–2.26). This association was more marked in those with both moderate-severe hearing loss (>40 dB HL) and presenting visual impairment, HR 1.84 (95% CI 1.19–2.86). Participants with either presenting visual impairment only or hearing impairment only, did not have an increased risk of mortality, HR 1.05 (95% CI 0.61–1.80) and HR 1.24 (95% CI 0.99–1.54), respectively. Concurrent best-corrected visual impairment and moderate-severe hearing loss was more strongly associated with mortality 10 years later, HR 2.19 (95% CI 1.20–4.03). Objectively measured DSI was an independent predictor of total mortality in older adults. DSI was associated with a risk of death greater than that of either vision loss only or hearing loss alone.

## Introduction

Impaired vision and hearing are common among older adults [1], [2] and can occur separately or in combination [3]. Visual loss impacts negatively on functional independence, mental health and cognition, and reduces quality of life, as well as increasing the need for support services [4]–[7]. Age-related hearing loss is more frequent and is associated with an increased risk of depression, and impairs quality of life and the ability to conduct activities of daily living, as well as leading to an increased reliance on community and informal supports [2], [8]–[13].

There are also prospective data to suggest that vision loss was associated with a greater mortality risk in older adults [14]–[16]. In a US study of 2520 adults aged between 65–84 years old, worse baseline acuity was associated with a 5% higher mortality rate. Recently, Karpa et al. [14] showed in the Blue Mountains Eye Study (BMES) of adults aged 49 years and over that the presence of presenting visual impairment increased the risk of all-cause mortality by 29%, after adjusting for potential confounders. Age-related hearing loss has also been shown to be associated with a greater risk of all-cause mortality in older adults [17]. In the Blue Mountains Hearing Study of adults aged 55 years and over, indirect path analyses showed that any level of hearing loss (>25 dB HL) was associated with increased all-cause mortality via three mediating variables: disability in walking, cognitive impairment and self-rated health [17].

There is a lack of population-based studies that have examined the association between the presence of objectively measured hearing and vision impairment, i.e. dual sensory impairment (DSI) and mortality risk. In the US National Health and Nutrition Examination Survey of 5444 adults aged 55–74 years, clinically confirmed DSI, however, was not found to be associated with an increased risk of mortality [18]. In an Italian study of 1140 adults aged 70–75 years, a significant association between DSI and 6-year total mortality was observed in men but not in women [19]. In this study, hearing loss was not measured objectively using pure-tone audiometry (i.e. ‘gold-standard’), but using the free-field whispered voice test. Finally, results from the US National Health Interview Survey of adults aged 18 years and older, showed that self-reported DSI was associated with a higher risk of all-cause mortality among men and women as compared to those reporting either visual impairment alone or hearing impairment alone [20], [21]. Many previous studies that have assessed the association between DSI and mortality had a relatively small number of participants with DSI at baseline and/or did not adequately adjust for mortality risk markers (e.g., cardiovascular diseases, diabetes, cognitive impairment and self-rated health) [18], [19], [22].

In the present study, we aimed to address previous gaps in knowledge, by examining the association between clinically confirmed DSI with 10-year mortality risk in a large cohort of adults aged 55 years and older, after adjusting for potential confounders such as self-rated health, walking disability and cognitive impairment, in addition to traditional mortality risk markers.

## Methods

### Study Population

The BMES is a population-based cohort study of common eye diseases and other health outcomes in a suburban Australian population located west of Sydney. Study methods and procedures have been described elsewhere [23]. Following a door-to-door census of the region, baseline examinations of 3654 residents aged >49 years were conducted during 1992-4 (BMES-1, 82.4% participation rate). Of the baseline participants, 2335 (75.1% of survivors) returned for 5-year follow-up examinations during 1997-9 (BMES-2), and 1952 participants (53.4% of the original cohort, or 76.6% of survivors) returned for 10-year follow-up examinations during 2002-4 (BMES-3). The study was approved by the Human Research Ethics Committee of the University of Sydney and was conducted adhering to the tenets of the Declaration of Helsinki. Signed informed consent was obtained from all the participants at each examination.

### Audiological Assessment

Pure-tone audiometry at both visits was performed by audiologists in sound-treated booths, using TDH-39 earphones and Madsen OB822 audiometers (Madsen Electronics, Denmark). Sound-proof rooms were set-up according to International Standards Organization protocol 8253-2. Bilateral hearing impairment was determined as the pure-tone average of audiometric hearing thresholds at 500, 1000, 2000, and 4000 Hz (PTA_0.5–4 kHz_) in the better ear, defining any hearing loss as PTA_0.5–4 kHz_ >25 dB HL; mild hearing loss as PTA_0.5–4 kHz_ >25–40 dB HL; and moderate to severe hearing loss as PTA_0.5–4 kHz_ >40 dB HL.

### Assessment of Visual Impairment

Monocular distance logMAR (logarithm of the minimum angle of resolution) visual acuity was measured with forced-choice procedures using the retroilluminated chart with automatic calibration to 85 cd/m^2^ (Vectorvision CSV-100 TM; Vectorvision Inc, Dayton, Ohio) according to the Early Treatment Diabetic Retinopathy Study protocol [23]. This was conducted with habitual correction (presenting visual acuity, with current eyeglasses, if worn) and after subjective refraction (best-corrected visual acuity). For each eye, visual acuity was recorded as the number of letters read correctly from 0 to 70. For the present study, any visual impairment was defined as presenting or best-corrected visual acuity of the better eye less than 39 letters (<20/40). DSI was defined as concurrent visual (either presenting or best-corrected) and hearing impairment, as determined using the above definitions.

### Information on Mortality

To identify and confirm persons who died after BMES-2, demographic information including surname, first and second names, sex and date of birth of the examined participants were cross-matched with Australian National Death Index (NDI) data for deaths, to December 2007. A probabilistic record linkage package was used, adopting a multiple pass procedure in which both data sets were grouped based on different characteristics (e.g., date of birth, name, sex) each time. Matches were divided into exact and non-exact. All non-exact matched records were examined manually and accepted if there was only one non-exact matched characteristic that was not critical. Information provided by family members during follow-up was also included if the participant was reported to have died on or before December 2007. The *International Classification of Diseases, Ninth Revision (ICD-9)* [24] and *International Statistical Classification of Diseases, 10^th^ Revision (ICD-10)* [25] cause of death codes were also obtained. The following cause-specific mortality codes were used in our analyses: coronary heart disease (ICD-9∶410.0–9, 411.0–8, 412, 414.0–9, and ICD-10: I21.0–9, I22.0–9, I23.0–8, I24.0–9, I25.0–9); stroke (ICD-9∶430.0–438.9 and ICD-10: I60.0-I69.9); or cancer (ICD-9∶140–208). The primary cause of death (i.e. death from any cause, stroke, coronary heart disease or cancer) was used in statistical modeling. The validity of Australian NDI data has been reported to have high sensitivity and specificity (93.7% and 100.0%, respectively) for all-cause mortality [26]. The census cut-off point for mortality was December 2007 (10-year follow up).

### Assessment of Covariates

A face-to-face interview with trained interviewers was conducted, and comprehensive data including information about medical history, hearing, demographic factors, socio-economic characteristics, lifestyle and health risk behaviour such as exercise, and smoking, were obtained from all participants. The medical history included cardiovascular or other systemic disease and associated risk factors, and medications used. A past history of angina, diabetes, myocardial infarction, and stroke was determined by responses to a question: “Has a doctor advised you that you have any of the following conditions?” Cognitive decline was assessed using the mini mental state examination (MMSE) questionnaire [27]. Participants with scores <24 were considered cognitively impaired. Self-rated health was assessed by asking: “For somebody your age, would you say your health is excellent, very good, good, fair, or poor?” Low self-rated health was defined as fair or poor.

Classification of hypertension was based on the 2003 World Health Organization/International Society of Hypertension guidelines [28]. Participants were classified as having hypertension stage 1 if systolic blood pressure was 140 to 159 mm Hg or if diastolic blood pressure was 90 to 99 mm Hg. Participants were classified as having hypertension stage 2 if they were previously diagnosed with hypertension and were using antihypertensive medications, if systolic blood pressure was 160 mm Hg or greater, or if diastolic blood pressure was 100 mm Hg or greater at examination. Body mass index was calculated as weight in kilograms divided by height in meters squared. Disability in walking at baseline was assessed as present if the participant was observed by a trained examiner to have walking difficulties or used walking aids or a wheelchair.

### Statistical Analysis

SAS statistical software (SAS Institute, Cary NC) version 9.1 was used for analyses. The association between single sensory impairment and DSI with mortality was examined using Cox regression models to estimate hazard ratios (HR) and 95% confidence intervals (CI). Multivariable regression models were first adjusted for age (entered as a continuous variable) and sex, and then further adjusted for confounders that were found to be significantly associated with mortality i.e. body mass index, systolic blood pressure, current smoking status, poor self-rated health, walking disability, presence of hypertension and/or diabetes, history of cancer, angina, stroke and/or acute myocardial infarction and cognitive impairment. We estimated the proportion surviving using the Kaplan Meier method. Kaplan-Meier survival curves are generated from the fitted Cox model using mean covariate values of age and sex.

## Results

**Figure 1:**
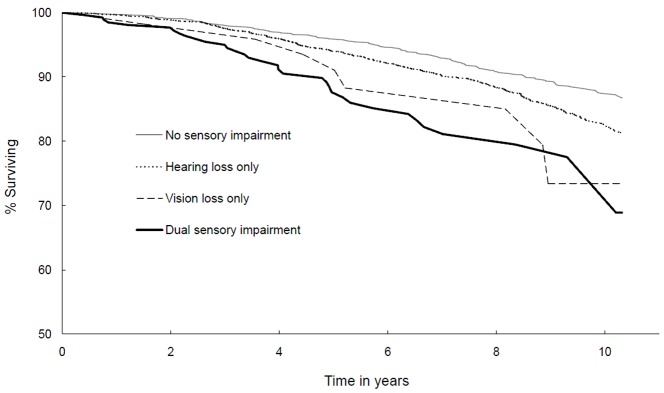
Age-sex adjusted Kaplan-Meier survival curves by presence of sensory impairment (presenting bilateral visual impairment and/or bilateral hearing impairment) among Blue Mountains Eye Study participants aged 55 years and older (n = 2806).

Table 1 shows the baseline characteristics of study participants with and without DSI. Those with DSI compared to those without DSI were more likely to be older, male, hypertensive and cognitively impaired, and also to have poorer self-rated health, walking disability, diabetes, stroke, angina, acute myocardial infarction, higher systolic BP and lower BMI (Table 1). We assessed the 10-year mortality risk in participants with presenting visual impairment (bilateral) and hearing impairment (Table 2). Among participants with presenting visual impairment (better eye) and bilateral hearing impairment at baseline, 64% had died 10 years later compared to 11% without any sensory loss, and 26% and 30% with only a vision or hearing loss, respectively. After multivariable adjustment, participants with DSI (i.e. presenting visual impairment and any level of hearing impairment) had a 62% increased risk of dying 10 years later compared to those without any sensory impairment, HR 1.62 (95% CI 1.16–2.26). This association was more marked in participants with both presenting visual impairment and moderate to severe hearing loss, multivariable-adjusted HR 1.84 (95% CI 1.19–2.86). Having a single sensory loss (i.e. either presenting visual impairment or any level of hearing loss) did not significantly increase the risk of mortality over 10 years. Figure 1 shows the 10-year survival of participants by the presence or absence of sensory loss. Persons with DSI had lower survival than those with a single sensory impairment (either presenting vision or hearing loss alone) or without any sensory impairment.

**Table 1:** Baseline characteristics of Blue Mountains Eye Study participants stratified by the presence of dual sensory impairment (n = 2812).

	Dual sensory impairment^a^		
Mortality risk marker	No(n = 1865)	Yes(n = 947)	p-value
Age, *yrs*	64.2 (7.8)	73.7 (8.4)	<0.0001
Male	745 (40.0)	471 (49.7)	<0.0001
Current smoker	187 (10.1)	86 (9.1)	0.43
Poor self-rated health	313 (16.9)	223 (23.7)	<0.0001
Body mass index, *kg/m^2^*	27.9 (4.7)	27.2 (4.7)	0.0004
Systolic bloodpressure, *mm Hg*	144.2 (21.0)	150.9 (22.3)	<0.0001
Walking disability	54 (2.9)	140 (14.8)	<0.0001
Diabetes	161 (8.6)	128 (13.5)	<0.0001
Hypertension^b^	1377 (74.0)	781 (82.6)	<0.0001
Stroke	52 (2.8)	67 (7.1)	<0.0001
Angina	141 (7.6)	150 (16.1)	<0.0001
Acute myocardial infarction	108 (5.8)	102 (11.0)	<0.0001
Cancer	208 (11.2)	106 (11.3)	0.94
Cognitive impairment^c^	20 (1.1)	59 (6.2)	<0.0001

Data are presented as mean (SD) and n (%), unless otherwise specified.

a Any hearing loss (>25 dB HL) and best-corrected visual impairment (<20/40).

b Hypertension Stage I –140/90–160/100; Stage II - >160/100 or treated.

c Cognitive impairment defined as mini-mental state examination score ≤24.

**Table 2:** All-cause mortality (over 10 years) in participants with bilateral hearing impairment and presenting visual impairment in the Blue Mountains Eye Study from 1997 to 2007 (n = 2806).

				Hazard ratio (95% confidence interval)		
Visual impairment (<20/40)	Hearing impairment (dB HL)		no. of deaths (%)		Model 1^a^	Model 2^b^
No visual impairment	≤25 (No hearing loss)		196 (10.9)		1.0 (reference)	1.0 (reference)
	>25 (Any hearing loss)		240 (30.2)		1.35 (1.10–1.65)	1.24 (0.99–1.54)
	>25-≤40 (Mild hearing loss)		153 (27.4)		1.33 (1.06–1.65)	1.27 (1.01–1.61)
	>40 (Moderate-severe hearing loss)		87 (37.0)		1.39 (1.06–1.83)	1.16 (0.87–1.56)
Presenting	≤25 (No hearing loss)		20 (25.6)		1.43 (0.90–1.65)	1.05 (0.61–1.80)
	>25 (Any hearing loss)		83 (63.9)		2.27 (1.70–3.05)	1.62 (1.16–2.26)
	>25-≤40 (Mild hearing loss)		44 (59.5)		2.10 (1.47–2.99)	1.46 (0.98–2.16)
	>40 (Moderate-severe hearing loss)		39 (69.6)		2.53 (1.74–3.68)	1.84 (1.19–2.86)

a Adjusted for age and sex.

b Further adjusted for body mass index, systolic blood pressure, current smoking status, poor self-rated health, walking disability, presence of hypertension and/or diabetes, history of cancer, angina, stroke and/or acute myocardial infarction and cognitive impairment.

Non-significant associations were observed between concurrent presenting visual impairment and hearing impairment at baseline with 10-year cause-specific mortality, after multivariable adjustment: coronary heart disease mortality - HR 1.47 (95% CI 0.83–2.58); stroke mortality - HR 1.05 (0.50–2.22); and cancer mortality – HR 1.79 (95% CI 0.99–3.23).

We also assessed the risk of mortality in participants with best-corrected visual impairment (bilateral) and hearing impairment (Table 3). Among participants with best corrected visual impairment and bilateral hearing impairment at baseline, 76% had died 10 years later compared to 11% without any sensory loss, and 40% and 33% with only a vision or hearing loss, respectively. After multivariable adjustment, having both best corrected visual impairment and moderate to severe hearing loss increased the mortality risk by two-fold, when compared to those without any sensory loss. Having any or mild hearing loss (but without best corrected visual impairment) was associated with an increased risk of dying of 29% and 32%, respectively.

**Table 3:** All-cause mortality (over 10 years) in participants with bilateral hearing impairment and best-corrected visual impairment in the Blue Mountains Eye Study from 1997 to 2007 (n = 2812).

				Hazard ratio (95% confidence interval)		
Visual impairment (<20/40)	Hearing impairment (dB HL)		no. of deaths (%)		Model 1^a^	Model 2^b^
No visual impairment	≤25 (No hearing loss)		208 (11.2)		1.0 (reference)	1.0 (reference)
	>25 (Any hearing loss)		290 (32.9)		1.41 (1.16–1.71)	1.29 (1.04–1.59)
	>25-≤40 (Mild hearing loss)		180 (29.6)		1.38 (1.12–1.71)	1.32 (1.06–1.66)
	>40 (Moderate-severe hearing loss)		110 (40.3)		1.46 (1.14–1.89)	1.22 (0.92–1.61)
Best corrected	≤25 (No hearing loss)		8 (40.0)		2.07 (1.01–4.22)	1.80 (0.79–4.12)
	>25 (Any hearing loss)		34 (75.6)		2.60 (1.74–3.90)	1.59 (1.00–2.52)
	>25–≤40 (Mild hearing loss)		17 (68.0)		2.17 (1.28–3.67)	1.20 (0.65–2.22)
	>40 (Moderate-severe hearing loss)		17 (85.0)		3.32 (1.95–5.63)	2.19 (1.20–4.03)

a Adjusted for age and sex.

b Further adjusted for body mass index, systolic blood pressure, current smoking status, poor self-rated health, walking disability, presence of hypertension and/or diabetes, history of cancer, angina, stroke and/or acute myocardial infarction and cognitive impairment.

## Discussion

To our best knowledge, this is the first population-based study to demonstrate that older adults with objectively measured DSI are at an increased risk of death from all causes compared to those without any sensory loss or a single sensory impairment. Specifically, participants with both presenting visual impairment (better eye) and bilateral hearing impairment at baseline had a 62% increased risk of dying 10 years later, independent of age, sex, self-rated health and the presence of known mortality markers. This association with mortality was more marked among older adults with concurrent moderate to severe hearing loss and any presenting or best-corrected vision loss.

Older adults in the BMES with clinically confirmed DSI compared to their counterparts without DSI had a 62% increased risk of total mortality. This finding is relatively similar to the HRs reported in a U.S. study: among men (HR 1.23 [95% CI 1.04–1.46]) and women (HR 1.63 [95% CI 1.37–1.93]) [20]. Also, in the Canadian National Population Health Survey [29], self-reported hearing impairment but not vision impairment were significantly associated with a greater risk of total mortality 12 years later, among 12,375 participants aged 18 and older. In our study, baseline DSI conferred a greater risk of dying 10 years later compared to having only presenting visual impairment or hearing impairment alone. This result suggests a potential interactive effect of DSI on survival, that is, the negative effects of vision are multiplied by the effects from hearing loss [12]. Our finding also concur with previous reports suggesting that the presence of more than one sensory impairment increases morbidity relative to single sensory impairments [21], [30]–[32].

We documented a gradient effect from the severity of DSI on mortality risk. Specifically, participants with concurrent moderate to severe hearing loss (>40 dB HL) and any visual impairment (<20/40) had a higher risk of dying 10 years later, than those with mild hearing loss (<25–40 dB HL) and any vision loss (particularly those with best-corrected visual impairment). This finding concurs with the US study by Lee et al. [21] which showed that moderate to severe concurrent hearing and vision impairment in women significantly increased their risk of mortality. This is not surprising, as with increasing level of measured hearing impairment the likelihood of communication difficulties also increases [33], which in turn can cause increasing social isolation [34] and also a higher likelihood of experiencing functional disability [13], factors that could negatively impact on life expectancy [35], [36].

It is hypothesized that the increased risk of mortality observed in persons with DSI is mediated by factors known to increase the risk of hearing and visual impairment in older adults (e.g., cardiovascular disease, hypertension and diabetes) [20], [37], [38]. However, in the BMES, a significant reduction in survival was observed in persons with DSI, even after controlling for cardiovascular risk factors (e.g., body mass index, smoking, hypertension) and events (e.g., angina, acute myocardial infarction), which supports findings from the study by Lam et al. [20] Moreover, we did not observe significant associations between DSI with 10-year coronary heart disease or stroke mortality. Therefore, these data together suggest that exposure to vascular risk factors are not likely to be the link between DSI and mortality risk, although, residual confounding from vascular risk factors cannot be fully discounted.

Alternatively, it is speculated that presence of DSI could be a marker for frailty (e.g., handgrip strength, peak expiratory flow), illness or possibly accelerated aging processes [21], [39]. Given that frailty is a strong predictor of mortality [40], the association between decreased vision and hearing and increased risk of dying is not unexpected. Additionally, a decline in psychosocial functioning could underlie the relationship between DSI and mortality risk. Visual impairment was shown to be associated with difficulty in performing ADL [41], social isolation [42] and depression [43]. Similarly, impaired hearing has also shown to negatively impact on participation in social activities [44], functional independence [13] and mental health [8]. Other potential mechanisms could be that both hearing and vision impairments are associated with a greater risk of accident and injury [45]–[48], which in turn could underlie the greater risk of mortality among older adults with DSI. However, we are unable to confirm this hypothesis as there were only around <1% of BMES participants who had died from injury (e.g. falls related) or accident, hence; we did not have sufficient study power to analyze these associations with DSI.

As sensory problems are common experiences within older age groups, they are often overlooked or dismissed [30]. Our study could have potential public health implications, as it suggests that identifying and targeting DSI in older adults could be a potentially useful strategy for preventing a decline in their life expectancy. Specifically, regular assessment of presence of DSI in older persons could lead to earlier detection and facilitation of rehabilitation and therapy that could reduce the negative impacts of DSI. Correction of visual and hearing impairment could improve survival [42], [49], hence, strategies including the provision of corrective lenses, hearing aids, assistive devices (e.g., magnifiers, listening devices) and rehabilitative services such as visual, auditory and communication training [21], [50] should be encouraged and/or implemented by clinicians in order to promote both longevity and an improved quality of life in their older sensory impaired patient.

The strengths of this study include the use of a representative cohort with a relatively high participation rate, the use of standardized audiometric and vision testing, with measures of sensory function at more than one point in time [51], and ascertainment of mortality and its causes using validated Australian National Death Index data. We previously showed that the door-to-door census which was used as a sampling frame for the BMES is less likely to be subject to selection bias [52]. This evidence together with the high response rate means that our door-to-door census can be regarded as being close to a gold standard as can realistically be achieved [53]. Despite the high internal validity of the BMES, we need to caution about the generalizability of our study findings. The study population is not a random sample of the wider Australian population. However, the findings from this study could be reasonably generalized to the Australian population over the age of 55, as it was previously observed that the demographic characteristics of this sample population are not markedly different from the Australian population apart from a lower proportion of immigrants [52]. Second, while we had robust data on a range of confounders, other unmeasured or unknown factors (e.g., lifestyle or societal factors) could have influenced our study findings. Finally, age was entered into the multivariable model assuming a linear relationship with mortality risk, hence, we cannot disregard the possibility that some of the effects of aging are being picked up by hearing impairment, vision impairment and/or other independent variables (e.g. walking disability).

In summary, we found that the presence of DSI independently predicted an increased risk of mortality in older adults. These findings emphasize to clinicians the importance of recognizing that older adults with concurrent vision and hearing loss are at an increased risk of mortality compared to their non-impaired counterparts or those with only a single sensory impairment. Public health strategies to encourage earlier identification of older adults with DSI and their appropriate referral to rehabilitative services and support could improve life expectancy in this vulnerable population.
